# The efficacy of task shifting interventions: A case of a selected psychiatric hospital in Zimbabwe

**DOI:** 10.4102/safp.v68i1.6236

**Published:** 2026-09-11

**Authors:** Nonhlanhla T. Ngwabi, Limkile Mpofu, Bheki T. Magunga

**Affiliations:** 1 Department of Environmental Health, Faculty of Human Sciences, University of Johannesburg, Johannesburg, South Africa; 2 The Research Division, Jaylee Group Institute, Pretoria, South Africa

**Keywords:** efficacy, intervention, mental health, psychiatric hospital, task shifting, task sharing

## Abstract

**Background:**

Task shifting is increasingly adopted to address healthcare worker (HCW) shortages in low-resource settings. Zimbabwe faces acute mental health workforce deficits, prompting redistribution of tasks to less specialised cadres. This study aimed to examine HCWs’ views on the effectiveness, implementation and consequences of task shifting in a major psychiatric hospital, and to identify training, policy, and system-level implications.

**Methods:**

A cross-sectional survey was conducted among 53 HCWs systematically sampled from a hospital workforce of about 500. Data were analysed using descriptive statistics, chi-square tests, correlation analysis and logistic regression in Statistical Package for the Social Sciences (SPSS) and Epi Info to assess associations between demographics, delegated tasks and perceived quality of care. Causality was not inferred because of the study design.

**Results:**

Participants reported confidence in performing delegated tasks but dissatisfaction with working conditions and concerns about mental health, training adequacy and scope clarity. Education level was significantly associated with specialised task delegation, while older workers were more likely to perform tasks without adequate training. Logistic regression indicated that participation in task shifting positively predicted perceived care quality, whereas participants with over 20 years of experience reported lower perceived quality.

**Conclusion:**

Task shifting is viewed as a feasible strategy to mitigate workforce shortages, but its success requires structured training, supervision, clear practice guidelines and improved working conditions. Policymakers should strengthen regulatory frameworks and prioritise training and mental health support.

**Contribution:**

This study provides evidence to inform policy, training and support interventions for the safe and effective scale-up of task shifting in Zimbabwean mental health services.

## Introduction

The mental health problem has become a global issue that is attributed to the growing burden of disease, calling for intervention strategies.^1^ In the words of Javadi and colleagues, intervention strategies would ‘strengthen the capacity of the health system to equitably and appropriately address the wide range of mental and neurological disorders’.^2^ Task shifting is another strategy that can strengthen the capacity of health systems. It is a process of delegation whereby tasks are moved, where appropriate, to less-specialised health workers to maximise the efficient utilisation of available healthcare workers (HCWs).^3^ This approach strengthens and expands the health workforce by shifting responsibilities from highly qualified health workers to those with less training and fewer qualifications.^3^ The strategy aims to optimise the population’s access to quality healthcare and services by decentralising and delegating tasks from healthcare professionals to less specialised HCWs. The World Health Organization (WHO) projects a global shortage of 12.9 million health workers by 2035,^4^ with the greatest deficits concentrated in low-income regions. Zimbabwe has experienced significant emigration of HCWs and deterioration of health system capacity, especially in mental healthcare.^5^

The healthcare sector employs task shifting and task sharing to enhance population access to quality healthcare services. This effort enables the healthcare sector to help the population access quality healthcare, thereby achieving Universal Health Coverage (UHC) and health-related sustainable development goals (SDGs).

The Zimbabwean healthcare system faces economic challenges that impact the people’s mental health.^6^ Psychiatric hospitals in Zimbabwe have a limited staff complement of psychiatrists, and this impacts the efficacy of healthcare service delivery as it endangers and leads to poor service delivery in various hospitals.^7^ The migration of skilled healthcare professionals leads to poor service.^6^ Such an exodus of healthcare professionals to greener pastures compromises the morale, task shifting and motivation of the remaining healthcare professionals to deliver good service in healthcare institutions. Researchers also lament that the unmet need for mental healthcare leads patients to turn to self-medication to compensate for the healthcare system’s poor service delivery.^8,9^ In his study, Nhapi^6^ confirms that psychiatric hospitals in Zimbabwe’s fees are heavily subsidised and that these hospitals’ allocations are insufficient for the rising demand from mental healthcare needs of the patients, leading them to resort to task shifting and task sharing.

Further still, several researchers have confirmed the brain drain and employee migration in African countries and urge institutions to devise strategies such as task shifting and task sharing to get the best results with the limited number of health workers available.^10,11^ Weinmann and Koesters^12^ further attest to the above assertion as they claim that, for mental health, the availability of services does not match population needs. At the same time, the WHO estimates the need for mental health workers to be 1.18 million for the mental health treatment gap to be closed.^2^

The following are successful examples of task shifting from resource-constrained countries. In Zimbabwe, for instance, Nyamayaro and colleagues confirm stepped care as a model for task-shifting for depression and that the clients are provided a low-intensity treatment that would only be intensified as required. The confirmation means that the interventions would be ‘feasible for non-specialists to deliver’ (p. 1).^13^ These researchers also report that the WHO developed a problem-solving therapy (PST) as part of its task-shifting toolkit for addressing depression in low to medium-income countries (LMIC).^13^ The toolkit formed part of the stepped-care guideline – the Mental Health Gap Action Plan.^14^ More still, Abas and colleagues also did a pilot study for the stepped care by task-shifting intervention for HIV patients with depression and low antiretroviral adherence.^15^ In this pilot study, lay community health workers were trained to deliver a first-level intervention to patients (six sessions of problem-solving cognitive behavioural therapy for depression and medication adherence). Thus, patients who had not benefited from the first-level intervention were preliminarily assessed to determine the urgency of their need for treatment and the nature of therapy so that they could be moved to more intensive treatment to be evaluated by trained clinicians for the potential provision of antidepressants and further counseling. Their findings revealed reduced depression and improved viral load suppression among intervention recipients compared with enhanced standard care.^15^

Another success story of task shifting for mental health intervention is highlighted by^16^ in Uganda. Here, the researchers carried out a randomised controlled trial in 10 clinics in Uganda.

They studied two task-shifting approaches to integrate depression treatment into HIV care. The trained nurses used a structured protocol for depression screening and treatment, while the trained primary care providers (PCP) used their clinical discretion (made use of an unstructured protocol).^16^ The results revealed successful screening. In 76% (nurses) and 80% (PCP) of cases, clinically depressed patients were prescribed antidepressants, while in 69% (nurses) and 56% (PCP) of cases, treated patients achieved complete remission of their depression at 65% (nurses) and 69% (PCP). Their results meant that the existing clinic staff (nurses, doctors) could provide quality depression care with limited training and supervision from available mental health specialists.

Further still, Murray and colleagues^17^ did a randomised controlled trial with lay counsellors in Zambia. These lay counsellors were to provide trauma-focused cognitive behavioural therapy (TF-CBT) to address trauma and stress-related symptoms among orphans and vulnerable children. This intervention by lay counsellors reduced the trauma and stress-related symptoms for orphans and vulnerable children.

The task-shifting approach has been successfully adopted in several African settings, including stepped care models for mental health in Zimbabwe, depression care innovations in Uganda, trauma management in Zambia and community-based mental health support programmes in Ethiopia, India, Nepal, South Africa and Uganda.^18^ They concluded that task-sharing mental health services is acceptable and feasible in low- to medium-income countries as long as key conditions are met. Such conditions include:

[*I*]ncreased numbers of human resources and better access to medications, ongoing structured, supportive supervision at the community and primary care levels, and adequate training and compensation for health workers involved in task sharing. (p. 33)^18^

Despite evidence of effectiveness, concerns persist regarding training adequacy, supervision, mental health burden, scope clarity and the impact of task shifting on the quality of care. In Zimbabwe, research has largely focused on disease-specific programmes rather than whole-facility task-shifting practices, leaving a gap in understanding how task shifting affects service delivery in psychiatric settings.

This study evaluated the perceived effectiveness, implementation and consequences of task shifting in a large psychiatric hospital in Zimbabwe, with a particular focus on training sufficiency, worker experience and perceived quality of care.

### Goal of the study

Task shifting can be a potential solution to mitigate the challenges of brain drain and improve the quality of service delivery in healthcare institutions.^19^ Therefore, the aim of this study was to assess the efficacy of task-shifting interventions for mental health in Zimbabwe. The findings could assist the development and implementation of support programmes to help healthcare practitioners use task-shifting and task-sharing as a strategy implemented by psychiatric hospitals.

## Research methods and design

### Study design

This study utilised a descriptive cross-sectional survey design to assess perceptions of the effectiveness, implementation and implications of task shifting in a psychiatric hospital in Zimbabwe. The cross-sectional design enabled comparison of multiple variables at a single point in time and was appropriate for evaluating associations without inferring causality.

### Setting

The study was conducted at a psychiatric hospital in Bulawayo, Zimbabwe. The institution provides a wide range of mental health services, including acute, subacute, forensic, rehabilitation and psychogeriatric care. The facility has a bed capacity of 708, with an average daily inpatient population exceeding 600 and an outpatient department catering to an average of 2400 patients per month.

### Study population

The study population consisted of HCWs at a psychiatric hospital. The inclusion criteria were HCWs whose roles involved being recipients of shifted task delegation, including nurses, doctors and nurse aides. Exclusion criteria included HCWs who were not delegated shift tasks, temporary HCWs on locum contracts and individuals on short-term academic attachments at the hospital.

### Sampling strategy

The estimated sample size was 52, calculated using the Centers for Disease Control and Prevention’s Epi Info statistical software, as shown in Figure 1.

**FIGURE 1:**
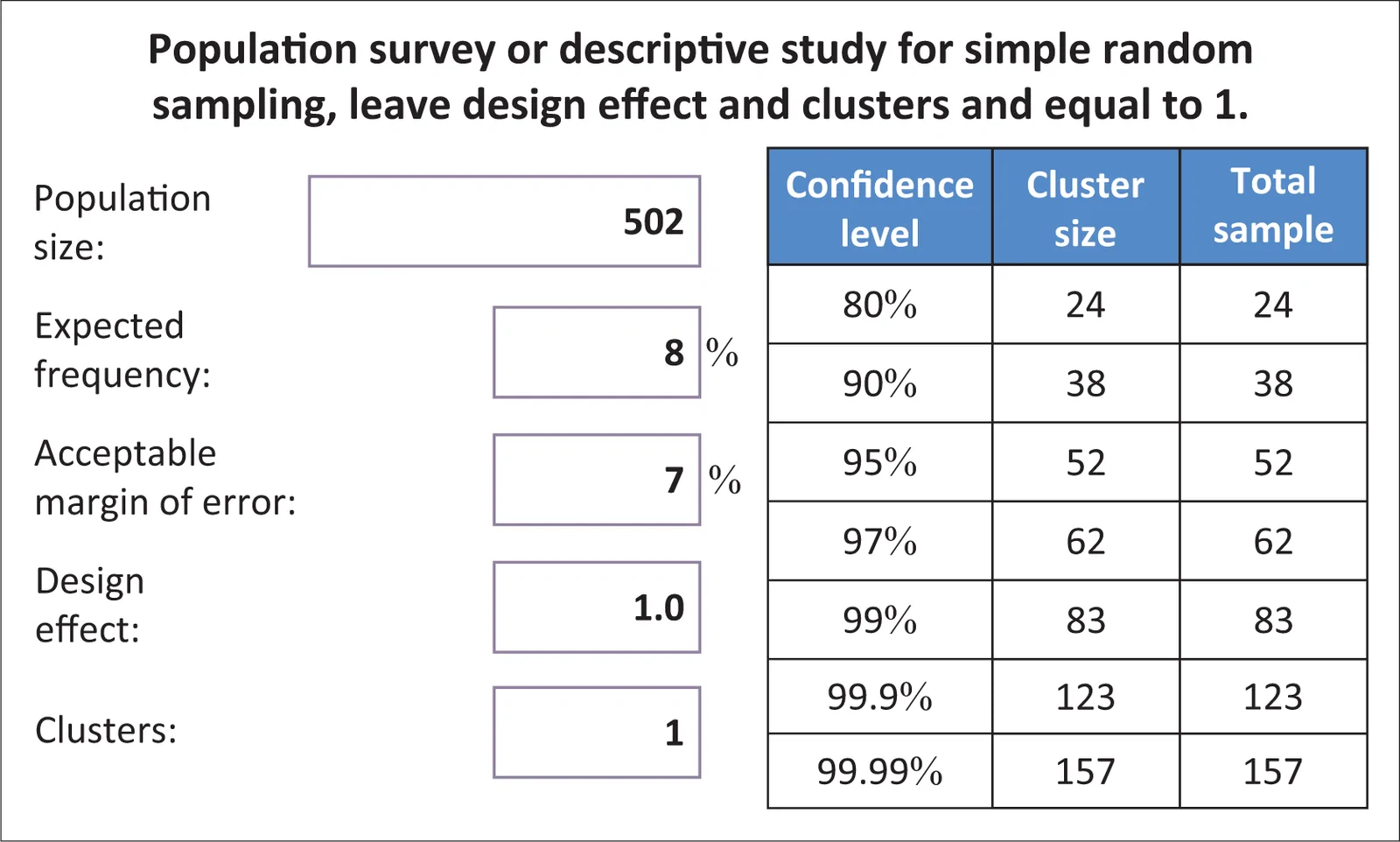
Sample size calculation.

Figure 1 illustrates how the sample size varies with the confidence level when conducting a population survey using simple random sampling. It uses a standard formula for estimating sample size for proportions, based on expected frequency, margin of error and population size. The calculator estimates the number of individuals the researchers need to survey from a population of 502 HCWs, assuming an expected frequency (prevalence) of 8%, which is the proportion of the population expected to exhibit the trait or condition. In Figure 1, the acceptable margin of error is ± 7%, the design effect is 1.0, and it assumes simple random sampling, with clusters of 1, indicating no clustering. To manually sample, the researchers employed a systematic sampling technique, where they selected the HCW at every tenth name on the randomly ordered population list. A total of 53 participants completed the questionnaire, providing a 100% usable response rate.

### Data collection

Data were collected using a structured online questionnaire designed and distributed via Google Forms. It consisted of multiple-choice questions, Likert-scale items, checkbox items and short responses. Content validity was ensured through expert review and pilot testing, achieving a content validity index (CVI) of 0.75. Test–retest reliability was assessed, yielding a high internal consistency with a correlation coefficient of 0.978.

The Google Forms research questionnaire tool supported automatic data collection and real-time response monitoring, allowing the researcher to track participation and analyse data promptly. Furthermore, the ability to distribute the questionnaire via email, WhatsApp Messenger or embedded links broadened the reach, ensuring a diverse and representative sample.

The questionnaire was pre-tested through a pilot study to ensure its validity and reliability. The pilot questionnaire was sent to HCWs in a setting similar to the anticipated study environment. Participants provided feedback on the content and layout of the questionnaire.

Approximately 67% of the participants expressed concerns about the length and complexity of some questions, suggesting that they could be summarised and worded better. The layout was generally well-received, but participants recommended more information on the purpose and scope of the questionnaire in the introduction. Responses were systematically organised in a Google Sheet, facilitating straightforward data manipulation and analysis.

Multiple strategies were employed to ensure the validity and reliability of this task-shifting study. Validity was addressed using a well-structured and pre-tested questionnaire aligned with the research objectives and theoretical frameworks established in previous studies. The questionnaire underwent a pilot test to identify and rectify any ambiguities, ensuring that questions were clear and relevant to the study’s aims. External validity was enhanced by employing a representative sample of HCWs in a public healthcare setting similar to the research setting, enabling the generalisation of the findings to the broader population. Reliability was ensured through standardised procedures for data collection, including training data collectors to ensure uniformity in administering the questionnaire and recording responses.

Test-retest reliability was assessed by administering the same questionnaire to a sample subset at two different points in time and calculating the correlation between the two sets of responses.

Data collection was conducted electronically. The questionnaire link was distributed to participants via institutional email and WhatsApp. Google Forms supported real-time monitoring, automatic collation and secure storage of responses. Participants completed the survey at their convenience, reducing response burden.

### Data analysis

Data were exported to Microsoft Excel for initial cleaning, coding and verification. This process involved checking for the accuracy, completeness and consistency of the responses. Statistical analyses were performed using SPSS and Epi Info.

### Descriptive and inferential statistics

Descriptive statistics were used to summarise demographic characteristics and task-shifting variables in order to illustrate the findings. Inferential statistics and their claims, complement the study, as they attempt to generalise beyond the sample.^20^ In our study, the effect sizes indicate the magnitude of task shifting, while the confidence intervals indicate the level of certainty. Together, they provide a richer interpretation than *p*-values alone, ensuring that both descriptive and inferential claims are properly contextualised and understood. Inferential statistics included Chi-Square tests to assess associations, correlation analysis to evaluate relationships between scope clarity and task ambiguity and logistic regression to identify predictors of perceived quality of care. No causality was inferred because of the cross-sectional design.

### Ethical considerations

The researchers adhered to all ethical principles regarding human subjects, and ethical approval was obtained from the Research and Ethics Committee of the University of Johannesburg, South Africa, prior to data collection. The ethical clearance number is REC-2247-2023. The study was conducted in accordance with ethical guidelines, ensuring that informed consent was obtained from all participants. This was achieved by including an informed consent statement on the first page of the form, which required participants to indicate their consent before accessing the questionnaire. Participation was voluntary, and informed consent was obtained from all participants. Confidentiality was maintained by anonymising all data, and responses were stored securely to prevent unauthorised access.

## Results

### Participant characteristics

Fifty-three HCWs participated in the study. Most participants were aged 25–44 years, and the majority were female. Professional diplomas were the most common qualification. The age distribution differed significantly from uniformity (*p* < 0.001), indicating a predominantly mid-career workforce. Educational level was significantly associated with task delegation patterns (*p* < 0.01). Gender and profession were not significantly associated with delegation patterns (see Table 1 for demographics and educational qualifications).

**TABLE 1:** Demographics and educational qualifications of participants.

Category	Most common	Proportion (%)	95% CI	Chi-square statistic	*P*-value
Age distribution (years)	35–44	35.85	23.11% – 48.58%	212	< 0.0010
Gender distribution	Woman	61.54	48.08% – 75.00%	2.15	0.7090
Highest level of education	Professional diploma	77.36	62.26% – 92.45%	37.65	0.0098
Profession	Psychiatric nurse	35.85	23.11% – 48.58%	22.26	0.5640
Professional qualification	Diploma	83.02	66.04% – 100.00%	29.47	0.0789

As visualised in Table 1, the age distribution was not uniform (χ^2^ = 212, *p* < 0.001), with the largest proportions of participants in the age groups 35–44 years (35.85%) and 25–34 years (28.30%), suggesting a workforce skewed towards mid-career professionals.

Gender distribution showed a non-significant deviation from uniformity (χ^2^ = 2.15, *p* = 0.709), with female participants representing 61.54% of the sample. Though numerically more prevalent, the difference was not statistically significant.

Educational attainment was significantly concentrated among holders of a professional diploma (77.36%; χ^2^ = 37.65, *p* = 0.0098), suggesting a dominant educational background in diploma-level training.

Regarding profession, the most frequently reported role was psychiatric nurse (35.85%). However, the chi-square test indicated no significant deviation from uniform distribution (χ^2^ = 22.26, *p* = 0.564), reflecting a relatively balanced professional representation across roles.

In terms of professional qualification, the data showed an over-representation of participants holding a diploma (83.02%), though the result did not reach statistical significance at the conventional 0.05 level (χ^2^ = 29.47, *p* = 0.079). This finding nonetheless indicates a homogeneity in formal qualification levels within the sample.

### Untrained task performance

Older HCWs were significantly more likely to perform untrained tasks (*p* = 0.0103). No significant associations were observed for gender, education or years of experience (see Table 2).

**TABLE 2:** Associations between demographics and untrained tasks of participants.

Variable	Chi-square statistic	*P*-value
Age	13.209	0.0103
Gender	2.249	0.1340
Education level	3.275	0.3510
Profession	5.562	0.3510
Professional qualifications	3.417	0.1800
Years of experience	2.838	0.8290

Additionally, while HCWs felt capable and supported in their task-shifting roles, there were significant concerns regarding mental health and the adequacy of training. Age was a significant factor in the likelihood of performing untrained tasks, with older HCWs more likely to engage in such activities. However, no significant associations were found between gender, years of experience, and the performance of untrained tasks (Table 2).

### Predictors of perceived quality of care

Task shifting was a significant predictor of perceived care quality (β = 4.48, *p* = 0.002). Participants with more than 20 years of experience reported lower perceived care quality (β = −2.42, *p* = 0.029), suggesting potential adaptation challenges or burnout. None of the other covariates, including professional qualification (diploma, degree, master’s, etc.) and years of experience, showed statistically significant associations (*p* > 0.05 for all), with the exception of those with more than 20 years of experience, who reported lower perceived quality (β = −2.42, *p* = 0.029) (see Table 3).

**TABLE 3:** Logistic regression predicting perceived quality of care.

Task shifting and background characteristics	Coef. (β)	Std. err.	*t*	*P* > |*t*|	95% CI	
Task shifting	4.48	1.35	3.303	0.002	1.74	7.21
Professional qualification: degree	-0.83	1.33	-0.623	0.537	-3.52	1.86
Professional qualification: diploma	-0.69	1.15	-0.603	0.550	-3.01	1.62
Professional qualification: masters	-2.40	1.86	-1.270	0.211	-6.21	1.41
Professional qualification: none	1.30	2.36	0.555	0.582	-3.46	6.07
Professional qualification: postgraduate degree	-0.48	1.99	-0.239	0.812	-4.50	3.54
Years of experience: 11–15	-0.53	0.91	-0.583	0.563	-2.36	1.30
Years of experience: 16–20	-0.79	1.00	-0.785	0.438	-2.80	1.23
Years of experience: 3–5	-0.69	0.88	-0.792	0.433	-2.46	1.07
Years of experience: 6–10	-1.08	0.82	-1.314	0.196	-2.74	0.58
Years of experience: less than one	-2.79	1.64	-1.695	0.098	-6.10	0.53
Years of experience: more than 20	-2.42	1.07	-2.264	0.029	-4.58	-0.26

### Dissatisfaction, mental health and scope clarity

Thirty per cent of participants reported dissatisfaction with their jobs, 50% with their working conditions and 40% with task-shifting support structures. Clarity of scope of practice showed a strong positive correlation with perceived confidence (*r* = 1.0) (see Table 4).

**TABLE 4:** Dissatisfaction analysis.

Aspect	Dissatisfied responses	Totalresponses	Percentage dissatisfied
Job satisfaction	30	100	30
Working conditions	50	100	50
Task shifting satisfaction	40	100	40

Figure 2 shows participants with more than 20 years of experience having a lower median quality of care rating. This indicates that, contrary to what might be expected, extensive experience does not necessarily correlate with higher perceived quality of care in this sample. The study’s findings suggest that task shifting can have both positive and negative impacts on healthcare delivery. While it enhances workforce efficiency and service coverage, it also raises concerns about job satisfaction and working conditions.

**FIGURE 2:**
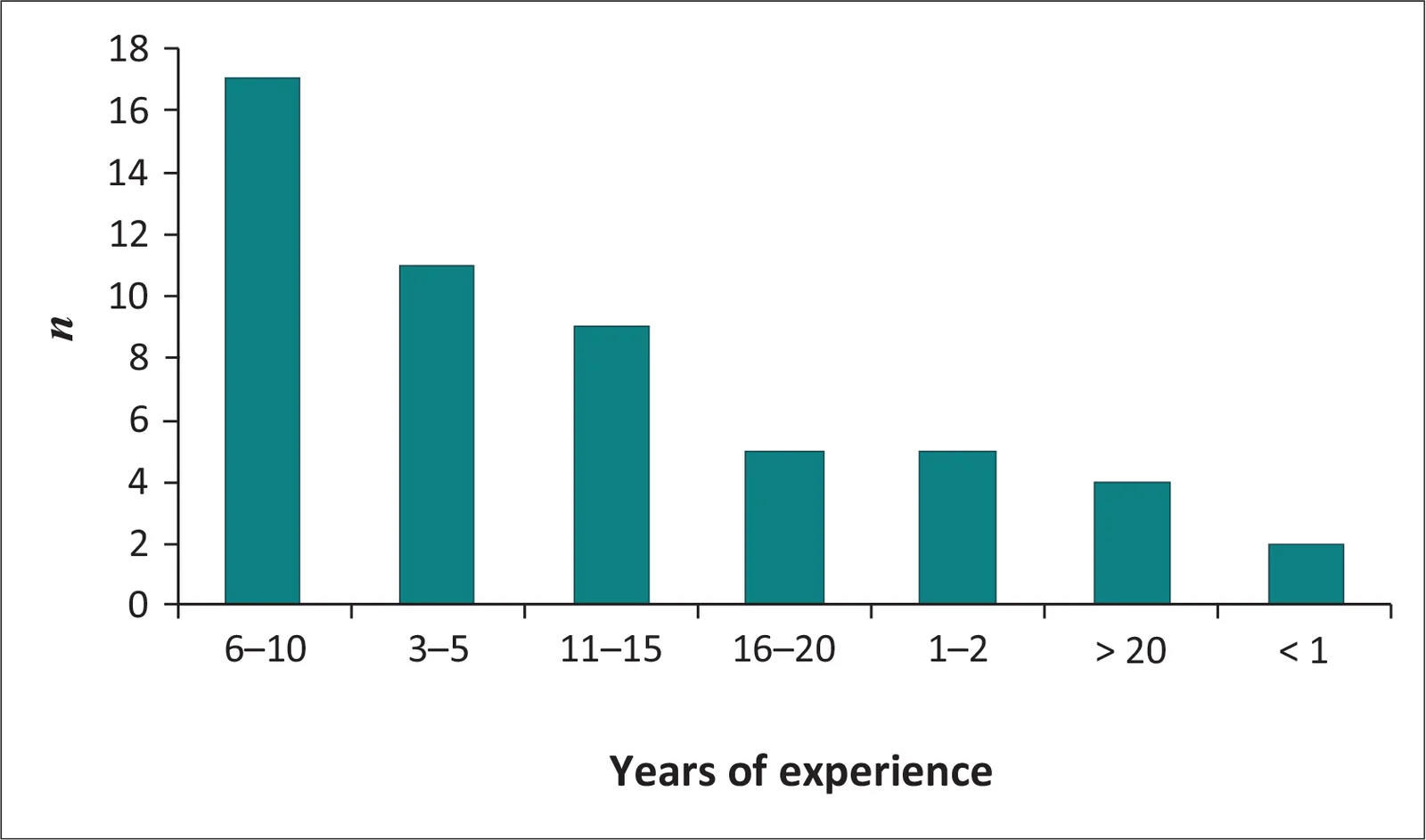
Distribution of years of experience.

Figure 3, on the other hand, illustrates the distribution of knowledge among the staff.

**FIGURE 3:**
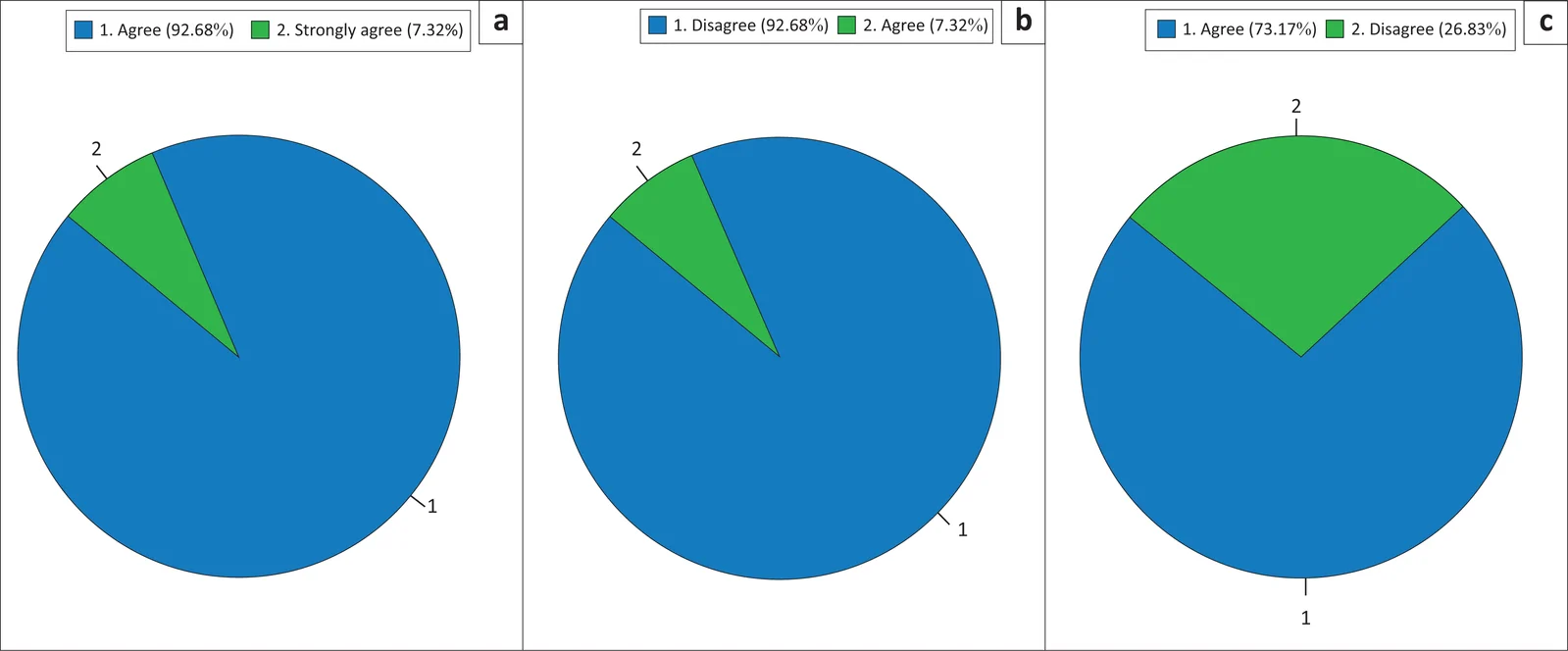
Illustration of participants’ (a) knowledge of scope, (b) scope information available, and (c) unavailability that contributes to undefined tasks.

Knowledge of scope is generally high, although it is inconsistently reported. Thus, the availability of scope information showed a strong positive correlation with knowledge (*r* = 1.0) but a negative correlation (*r* = –0.25) with the perception that unavailability leads to undefined tasks. The perceived unavailability of scope information was moderately associated with dissatisfaction and concerns about task ambiguity, especially in contexts where formal guidelines are lacking.

A detailed illustration of the distribution of untrained task performance is shown in Figure 4.

**FIGURE 4:**
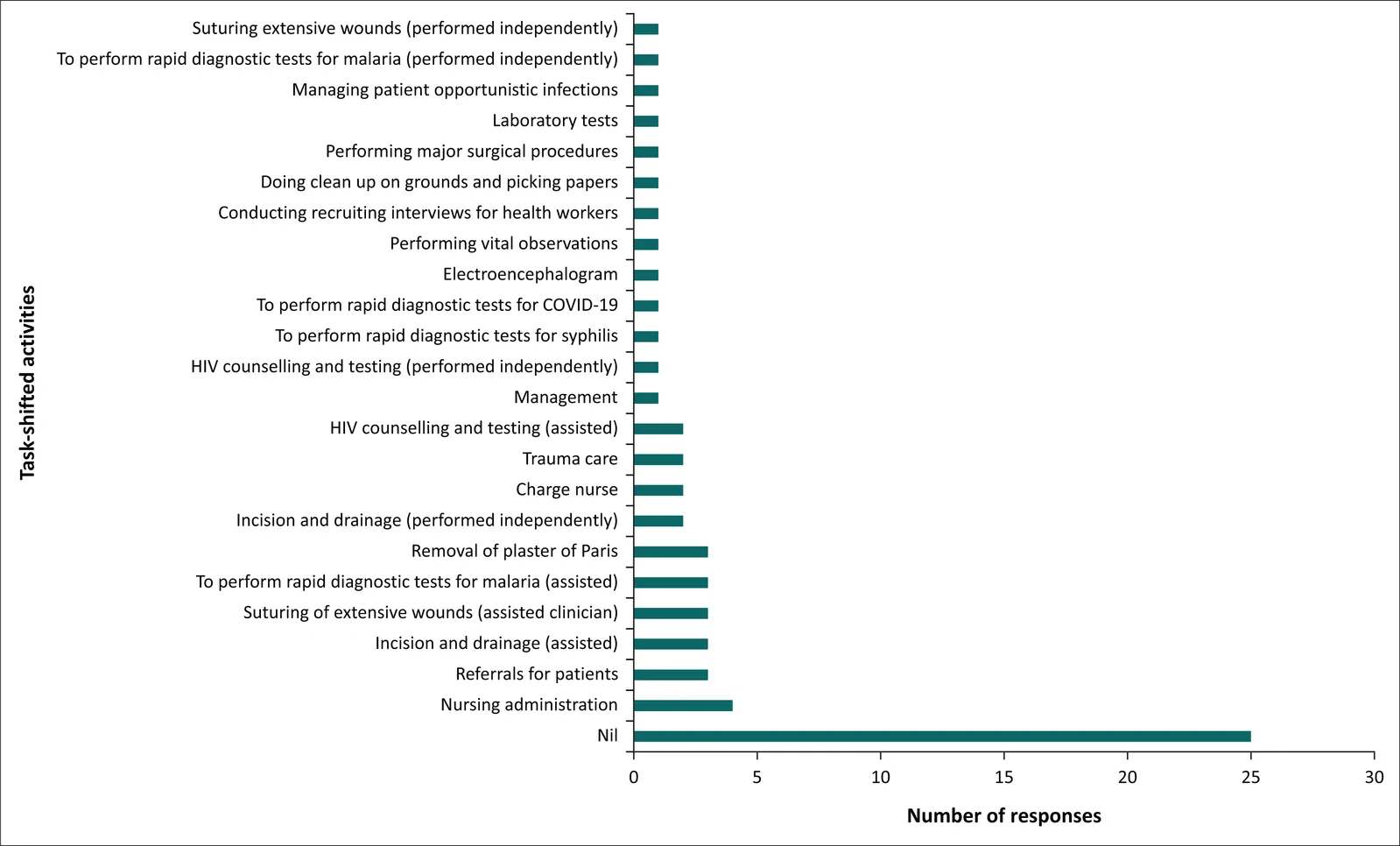
Distribution of untrained task performed by participants.

## Discussion

The study demonstrates that task shifting is firmly embedded within clinical operations at the psychiatric hospital, with HCWs expressing strong confidence in performing delegated tasks. Despite this integration, the scale-up of task-shifting strategies for mental health across Africa remains constrained, largely because of the persistent brain drain of professional personnel who migrate in search of better opportunities.^12^ This exodus of skilled staff leaves health systems vulnerable, with the remaining workforce often overwhelmed, unmotivated and demoralised.^21^

Our findings highlight that task shifting enables mental health specialists to dedicate their expertise to complex cases without compromising the quality of care while simultaneously improving efficiency. Similar conclusions were drawn by Spedding, Stein, and Sorsdahl,^22^ who reported broad stakeholder acceptance of task shifting in South Africa. Likewise, Weinmann and Koesters^12^ found that the involvement of lay providers in HIV care allowed specialists to concentrate their efforts on advanced clinical challenges. Collectively, these results reinforce the growing evidence that task shifting is emerging as a promising health systems strategy.^23^

Nonetheless, this study also surfaces critical concerns. Participants noted challenges related to workload, mental health strain, workforce shortages and insufficient training. Education level emerged as a strong predictor of specialised task delegation, while older workers were more likely to perform tasks without formal training. Logistic regression analysis further revealed that engagement in task shifting was positively associated with perceived quality of care, although those with more than 20 years of experience reported lower perceived care quality. These nuanced findings underscore the importance of tailoring task-shifting interventions to the demographics of the workforce.

Van Schalkwyk and colleagues argue that the ‘persistence of rigid professional boundaries can be challenged’ (p. 1379),^11^ a perspective that is echoed in our results. Consistent with studies from Zimbabwe, Uganda, Zambia, Ethiopia and South Africa, our findings confirm that task shifting can expand access to mental health and HIV services while alleviating staffing shortages.^15,17,18,24^ However, prior research has also identified persistent barriers, including inadequate supervision, role ambiguity and the emotional burden placed on frontline workers.^25^

Evidence from multiple studies suggests that successful task shifting requires enabling conditions such as regular supervision, adequate resources and medicines, structured training and ongoing professional development through in-service programmes.^14,24,26^ Our study reinforces this view, emphasising that continuous professional development and robust support systems are essential for sustaining high standards of care. These results align with global evidence demonstrating that task shifting enhances service delivery when supported by strong training structures and clear scope-of-practice guidelines.^22^

### Implications for policy and practice

The findings underscore the urgent need for policies that formalise task-shifting frameworks through standardised training curricula, clear supervision models and well-defined scopes of practice. Such formalisation would safeguard the quality of care and ensure that delegated responsibilities are performed safely and effectively.^23^ Strengthening supervision structures is particularly critical, as evidence shows that consistent oversight enhances accountability and supports safe delegation practices.^27^

Equally important are investments in continuing professional development, which can bolster HCWs’ confidence, improve performance and sustain motivation in resource-constrained settings.^25^ Improving working conditions and embedding mental health support systems for HCWs should also be prioritised, given the documented risks of burnout, emotional strain and demoralisation among frontline staff engaged in task-shifting roles.^24^

Taken together, these implications highlight that task shifting is not merely a stopgap measure but a strategic health systems intervention. When supported by robust training, supervision and workforce wellbeing initiatives, task shifting can enhance service delivery, optimise specialist expertise and strengthen the resilience of health systems facing chronic human resource shortages.^22^

### Strengths

A key strength of this study lies in the use of systematic sampling, which enhanced representativeness and reduced selection bias, thereby strengthening the validity of the findings.^28^ The application of validated data collection tools with demonstrated reliability further ensured consistency and accuracy in measurement.^29^ In addition, the study employed appropriate statistical methods for a cross-sectional design, allowing for robust analysis of associations within the dataset.^30^

### Limitations

However, several limitations must be acknowledged. Firstly, the reliance on self-reported data introduces the possibility of recall bias and social desirability bias, which may affect the accuracy of responses.^31^ Secondly, the single-site setting limits external validity and may reduce the generalisability of findings to other contexts, particularly across diverse health systems in Africa. Thirdly, the cross-sectional natureof the study precludes causal inference, restricting conclusions to associations rather than directional effects.^32^

Future research should therefore adopt multi-site designs to capture broader contextual variations and enhance generalisability. Moreover, mixed-methods approaches could provide deeper insights into the implementation challenges and workforce perceptions surrounding task shifting, combining quantitative rigour with qualitative exploration of lived experiences.^33^ Such designs would strengthen the evidence base and inform policy frameworks for scaling task-shifting strategies in mental healthcare.

## Conclusion

Task shifting remains a critical strategic response to the severe shortage of mental health professionals in Zimbabwe and across sub-Saharan Africa. This study demonstrates that while HCWs express strong confidence in performing delegated roles, the sustainability and effectiveness of task shifting depend on structured training, clearly defined scopes of practice and robust supervision systems. Addressing gaps in working conditions and embedding psychosocial support for HCWs are equally vital to safeguarding both staff wellbeing and the quality of patient care.

The findings further highlight the need for policy frameworks that institutionalise task shifting through competency-based training, strengthened supervision, and regulatory safeguards to ensure safe and effective delegation. Investments in workforce development and mental health support structures will be essential to sustaining long-term impact. Future research employing multi-site designs and mixed-methods approaches can provide deeper insights into system-level challenges and opportunities, thereby informing the optimisation of task-shifting models in psychiatric and broader healthcare settings.
